# Supplementary material for: RBM20 isoform regulation by independent transcription start sites adapts alternative splicing in development and disease
Source: Nat Commun. 2026 May 23;17:4607. doi: 10.1038/s41467-026-73230-w (PMC13198548; doi:10.1038/s41467-026-73230-w)
Supplement: Supplementary file 1 — Supplementary Information [file 41467_2026_73230_MOESM1_ESM.pdf]

Supplementary Data and Tables

Differential gene expression and alternative splicing data related to Figure 3:

- Supplementary Data 1 Differential Gene Expression analysis of RBM20 lacZ and RRM mice.
- Supplementary Data 2 Differential Splicing analysis of RBM20 lacZ and RRM mice.

Alternative splicing data related to Figure 5:

- Supplementary Data 3 Differential Splicing analysis of SHR vs BN rat.
- Supplementary Data 4 Differential Splicing analysis in human HCM.
- Supplementary Data 5 Differential Splicing analysis in human DCM.
- Supplementary Data 6 RBM20 target genes

Supplementary Table 1: Primers for Sybr green or TaqMan qRT-PCR

Primer	sequence	info
18S for	CGCCGCTAGAGGTGAAATTC	normalization
18S rev	TGGGCAAATGCTTTCGCTC	normalization
Rbm20-ex1-F	GGAGCAACCCGACAGAGATG	long isoform
Rbm20-Ex2-R	GTGAGCTGAGCCTGTATCTGG	long isoform
RBM20 EST f2	GTACATGTGCGACACCCAAA	short isoform
Rbm20-Ex2-R	ACCGTGGCCGCTGCAGTGTTG	short isoform
Rbm20-Ex2-F	CCTGCCTTTGGGTCTCGGCTTAAC	all isoforms
Rbm20-Ex3-R	CCCTTTCACATGTAGCTCCCAGTC	all isoforms
mRBM20_Ex8f	CTGCTATCATCCAAGACATCC	all isoforms/ normalization
mRBM20_Ex9r	GAGTGATCGGCTCATTGG	all isoforms/ normalization
RBM20	Hs01098240_m1	Transfected RBM20 detection
Human 18S rRNA	4310893E	real time PCR, control

Supplementary Table 2: Antibodies used for Western blot

Antibody	Species	Dilution	Manufacturer	ID
RBM20	rabbit	1:500	Own production[1]	-
Vinculin	mouse	1:1000	Sigma	V9131
Anti-a-Tubulin (DM1A)	mouse	1:5000	Calbiochem	CP06
C-Myc	mouse	1:1000	Invitrogen	13-2500

Supplementary Table 3. Antibodies for immunofluorescence staining

Antibody	Species	Dilution	Manufacturer	ID
RBM20	rabbit	1:50	Own production[1]	
α-Actinin EA53	mouse	1:200	Sigma	A7811
U2AF65	mouse	1:100	Sigma	U4758
SF2/ASF	mouse	1:100	Invitrogen	32-4600
Alexa Fluor 488	goat	1:1000	Thermo Fisher	A11034
Alexa Fluor 568	goat	1:1000	Thermo Fisher	A11031

Supplementary Table 4. Exon coordinates of *RBM20*

species	chr	start	end	exon name	genome version
**human**	10	110644336	110644645	E1	GRCh38.111
**human**	10	110764615	110764825	E1B	GRCh38.111
**mouse**	19	53665737	53666023	E1	GRCm39.111
**mouse**	19	53781746	53781877	E1B	GRCm39.111
**mouse**	19	53649422	53649536	E1A	GRCm39.111
**rat**	1	252683771	252684052	E1	RatBN7.2.111
**rat**	1	252814564	252814837	E1B	RatBN7.2.111

Supplementary Figures:

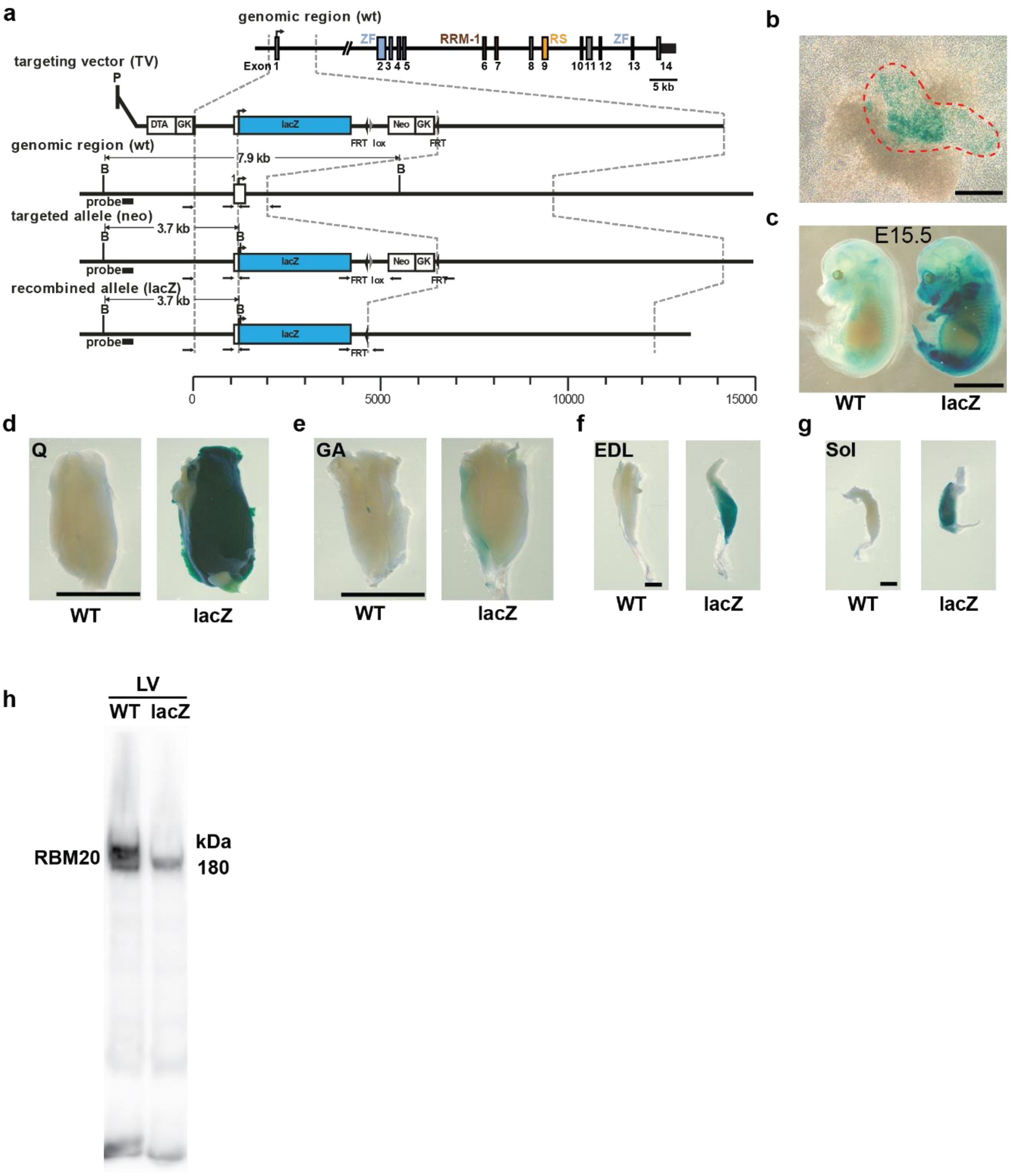

Supplementary Figure 1: Generation and validation of RBM20 knockout by introducing a *lacZ* cassette in-frame into exon 1**.**

a) Targeting strategy, exons colored by encoded domains, 5'UTR in black, *lacZ* coding region in blue. **b)** ES cell-derived cardiomyocytes, beating area marked by dotted red line also display beta-galactosidase staining. Independent observations on five colonies from one experiment **c)** Embryo at E15.5 displays beta-galactosidase staining in heart and developing skeletal muscle in RBM20 lacZ-HOM animals. Two replications on two independent sets of animals. Beta-galactosidase staining **d)** quadriceps (Q), **e)** gastrocnemius (GA), **f)** extensor digitorum longus (EDL), and **g)** soleus (Sol) muscles Analysis of four sets of tissues in three independent experiments. Scale bar in B 0.2 mm; C 5 mm; D and E 5 mm; F and G 1 mm. Zink finger domain (ZF), RNA-recognition- motive (RRM),

arginine-serine-rich domain (RS), FLP-recombination site (FRT), Cre-recombination site (lox), neomycin resistance cassette (Neo), GK Promoter (GK), diphtheria toxin cassette (DTA). h) full length Western blot of figure 1f RBM20 Western blot indicates long and short isoforms in WT hearts, while in the lacZ mice only the shorter one is detected.

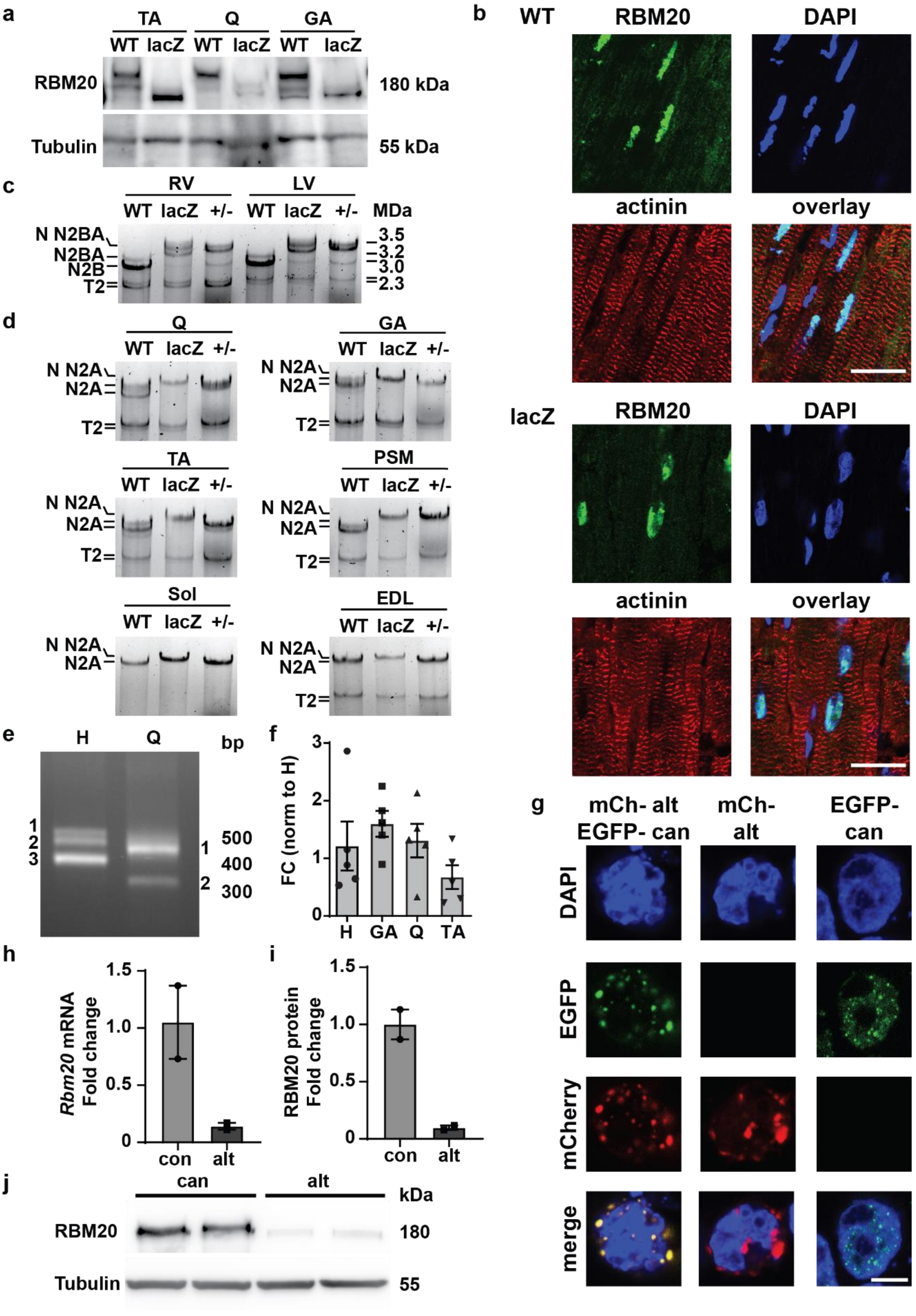

Supplementary Figure 2: RBM20 protein levels and titin isoform adaptation.

a) RBM20 Western blot of TA, Q and GA muscles indicates expression of a shorter RBM20 isoform. **b)** RBM20 localizes to the nucleus in WT and lacZ HOM hearts. Two independent experiments on two independent sets of tissue. **c)** Changes in titin splicing in different cardiac regions, Atr = atrium; RV right ventricle; LV left ventricle and **d)** skeletal muscles; Q= Quadriceps; GA= Gastrocnemius muscle; TA= Tibialis anterior; PSM= Psoas mayor; Sol= Soleus; EDL= Extensor digitorum longus. Two independent experiments on two sets of tissue. **e)** Additional transcription start sites found by 5'RACE PCR in mouse heart (H) and quadriceps (Q). Numbers indicate the bands cut for sequencing. **f)** Expression of shorter *Rbm20* isoforms analyzed by qRT-PCR (n = 5). Norm to H = normalized to heart. **g)** Colocalization of EGFP-RBM20 canonical (EGFP-can) and mCherry RBM20 alternative (mCh-alt) isoform in transiently transfected HEK cells. Two independent experiments. **h)** Reduced expression of RBM20 alternative mRNA and **i)** reduced RBM20 alternative protein in HEK-EBNA transfected cells (n=2). **j)** Western blot of RBM20 transfected HEK-EBNA cells 48h after transfection. Two independent experiments. f), h), i) Mean +/- SEM. FC = fold change, can = canonical, alt = alternative isoform. Scale bar in b) 20 µm in g) 5µm.

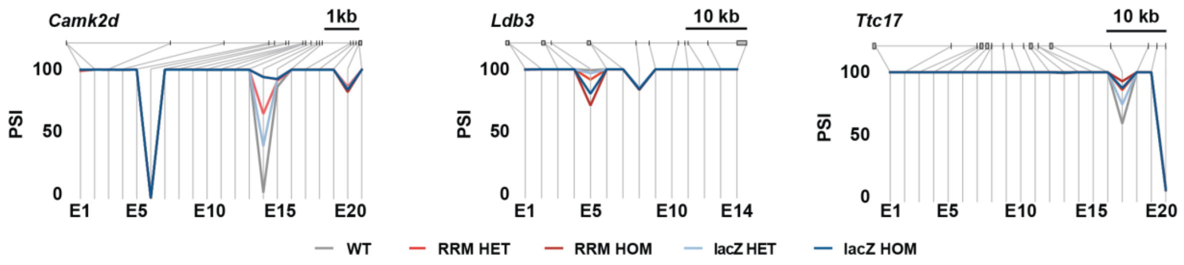

Supplementary Figure 3: Differential gene splicing in the RBM20 RRM KO and LacZ animals.

Percent spliced-in (PSI) plots of *Camk2d*, *Ldb3* and *Ttc17*. Transcript structure is represented above the trace. Exons are marked by boxes and introns by grey lines. Generated from mRNA-seq data. n = 3 for WT and RRM HET, n = 4 for RRM HOM, lacZ HET and lacZ HOM.

a

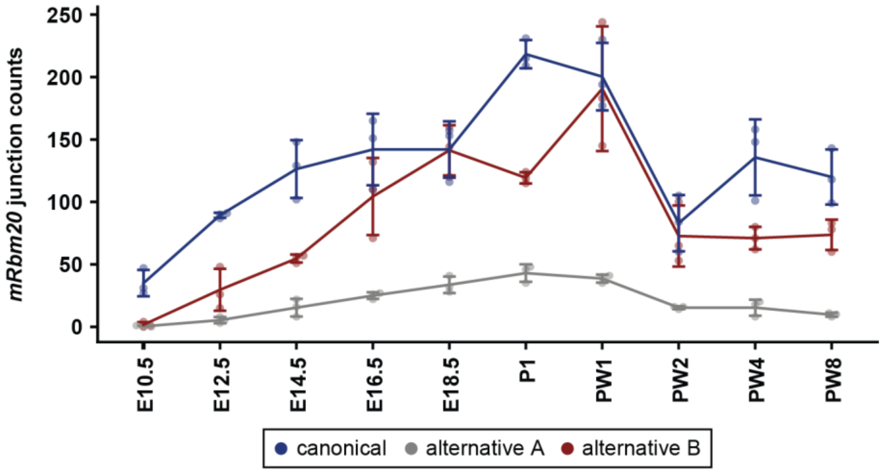

b

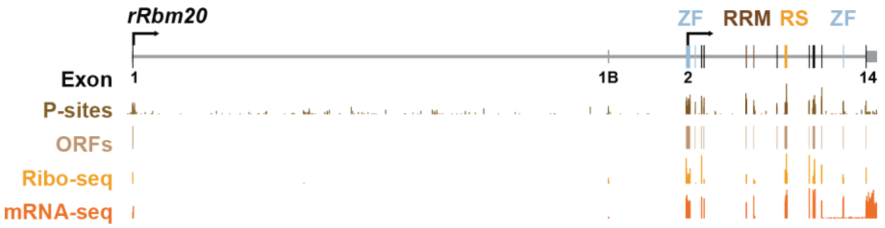

Supplementary Figure 4: Rbm20 expression during mouse development and rat Rbm20 Ribo-seq analysis

a) Expression of mouse *Rbm20* isoforms in development time line. Rbm20 junction reads spanning from exon 1 to exon 2 in each developmental stage sample based on reads mapping to the canonical (blue), alternative B (red), or alternative A (grey) transcription start. Mean ± SD, n = 3. **b)** Rat *Rbm20* gene structure, P-sites, open reading frames (ORF), mRNA- and Ribo-seq data. ZF: zinc finger, RRM: RNA recognition motif, RS: arginine and serine rich region.

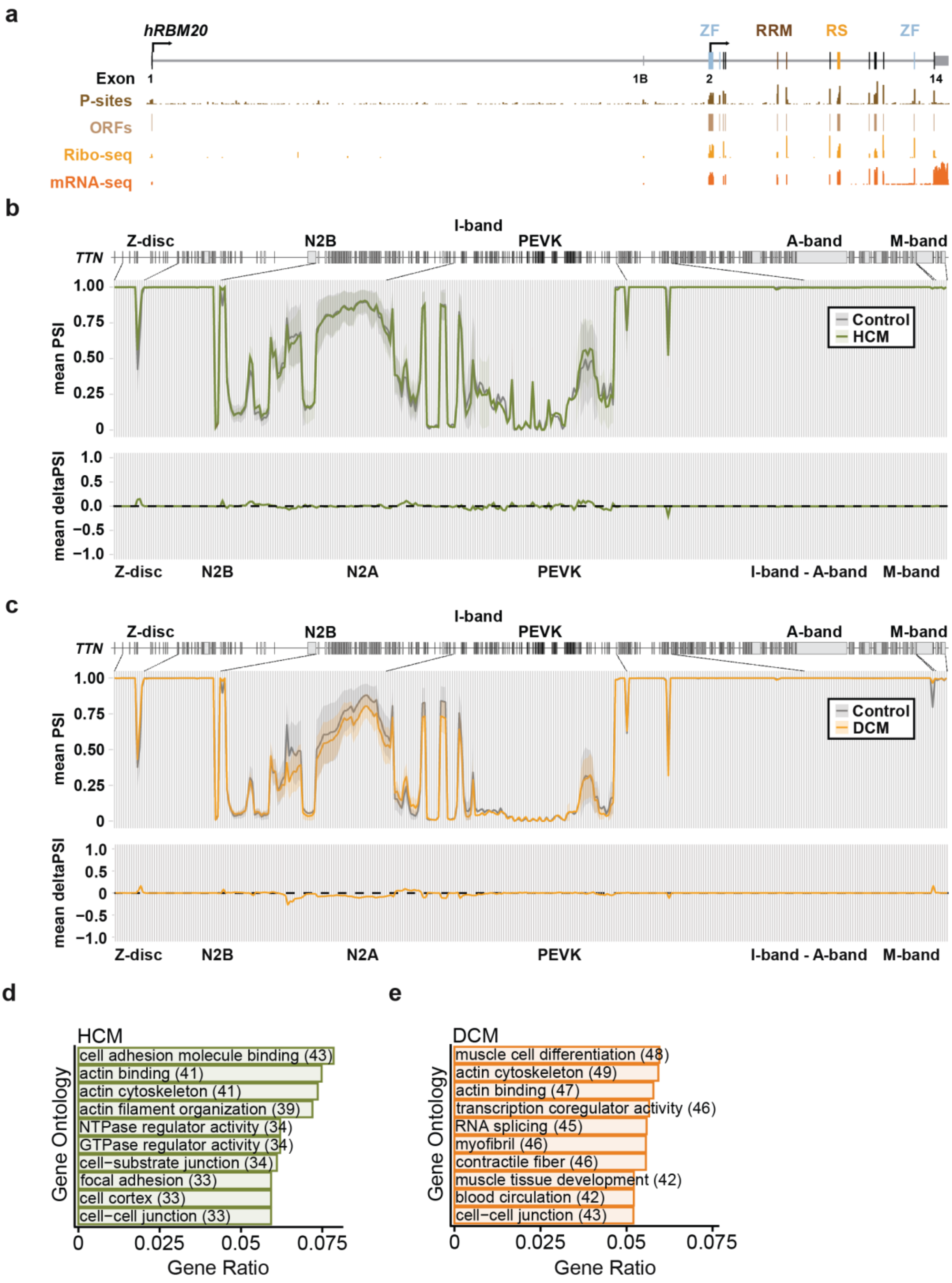

Supplementary Figure 5: Analysis of human *RBM20* Ribo-seq and titin splicing in cardiac disease

a) Human *RBM20* gene structure*,* P-sites, open reading frames (ORF), mRNA- and Ribo-seq data. ZF: zinc finger, RRM: RNA recognition motif, RS: arginine and serine rich region. **b)** Percent spliced-in (PSI) and mean delta PSI plots of *TTN* in HCM[2] (Control n = 23, HCM n = 97) or **c)** DCM[3] patients (Control n = 105, DCM n = 96). Transcript structure is represented above the trace. Exons are marked

by boxes and introns by grey lines. HCM titin splicing is shown in green, DCM titin splicing is shown in orange, control samples are shown in grey. Mean ± SD. **d,** Gene Ontology of genes differentially spliced in HCM and **e)** DCM.

Supplementary References

1. Guo, W. *et al.* RBM20, a gene for hereditary cardiomyopathy, regulates titin splicing. *Nat. Med.* **18**, 766–773 (2012).
2. Garmany, R. *et al.* A multi-omics atlas of sex-specific differences in obstructive hypertrophic cardiomyopathy. *J. Mol. Cell. Cardiol.* **196**, 26–34 (2024).
3. Heinig, M. *et al.* Natural genetic variation of the cardiac transcriptome in non-diseased donors and patients with dilated cardiomyopathy. *Genome Biol.* **18**, 170 (2017).
