# Supplementary material for: RBM20 isoform regulation by independent transcription start sites adapts alternative splicing in development and disease
Source: Nat Commun. 2026 May 23;17:4607. doi: 10.1038/s41467-026-73230-w (PMC13198548; doi:10.1038/s41467-026-73230-w)
Supplement: Supplementary file 2 — Description of Additional Supplementary Files [file 41467_2026_73230_MOESM2_ESM.pdf]

Description of Additional Supplementary Files

Differential gene expression and alternative splicing data related to Figure 3:

- Supplementary Data 1: Differential Gene Expression analysis of RBM20 lacZ and RRM mice.
- Supplementary Data 2: Differential Splicing analysis of RBM20 lacZ and RRM mice.

Alternative splicing data related to Figure 5:

- Supplementary Data 3: Differential Splicing analysis of SHR vs BN rat.
- Supplementary Data 4: Differential Splicing analysis in human HCM.
- Supplementary Data 5: Differential Splicing analysis in human DCM.
- Supplementary Data 6: RBM20 target genes
