# Supplementary material for: RBM20 isoform regulation by independent transcription start sites adapts alternative splicing in development and disease
Source: Nat Commun. 2026 May 23;17:4607. doi: 10.1038/s41467-026-73230-w (PMC13198548; doi:10.1038/s41467-026-73230-w)
Supplement: Supplementary file 9 — Reporting Summary [file 41467_2026_73230_MOESM9_ESM.pdf]

nature portfolio

Corresponding author(s): Michael Gotthardt

Last updated by author(s): Apr 26, 2026

Reporting Summary

Nature Portfolio wishes to improve the reproducibility of the work that we publish. This form provides structure for consistency and transparency in reporting. For further information on Nature Portfolio policies, see our Editorial Policies and the Editorial Policy Checklist.

Statistics

For all statistical analyses, confirm that the following items are present in the figure legend, table legend, main text, or Methods section.

n/a	Confirmed	
☐	☒	The exact sample size (*n*) for each experimental group/condition, given as a discrete number and unit of measurement
☐	☒	A statement on whether measurements were taken from distinct samples or whether the same sample was measured repeatedly
☐	☒	The statistical test(s) used AND whether they are one- or two-sided<br>*Only common tests should be described solely by name; describe more complex techniques in the Methods section.*
☐	☒	A description of all covariates tested
☐	☒	A description of any assumptions or corrections, such as tests of normality and adjustment for multiple comparisons
☐	☒	A full description of the statistical parameters including central tendency (e.g. means) or other basic estimates (e.g. regression coefficient) AND variation (e.g. standard deviation) or associated estimates of uncertainty (e.g. confidence intervals)
☐	☒	For null hypothesis testing, the test statistic (e.g. *F*, *t*, *r*) with confidence intervals, effect sizes, degrees of freedom and *P* value noted<br>*Give P values as exact values whenever suitable.*
☒	☐	For Bayesian analysis, information on the choice of priors and Markov chain Monte Carlo settings
☒	☐	For hierarchical and complex designs, identification of the appropriate level for tests and full reporting of outcomes
☐	☒	Estimates of effect sizes (e.g. Cohen's *d*, Pearson's *r*), indicating how they were calculated

Our web collection on statistics for biologists contains articles on many of the points above.

Software and code

Policy information about availability of computer code

Data collection	https://www.ebi.ac.uk/ena/browser/view/PRJEB89457
Data analysis	Data analysis scripts are provided on github (https://github.com/MitchGotthardt/GotthardtLab/tree/main). Software and tools used: TrimGalore v0.6.6, AltAnalyze v2.1.4.3, Leafcutter v0.2.9, Cytoscape v.3.9.0, ClueGO v2.5.9, Venn Diagram Plotter v1.6, Integrative Genome Viewer v2.16, fastp v0.23.2, STAR v2.7.8a, Salmon v1.10.1, tximport v1.30.0, DESeq2 v1.42.1, apeglm v1.24.0, PSI calculation scripts from https://github.com/MIAOKUI/PSI, rMATS-turbo v4.3.0, clusterProfiler v4.10.1, org.Hs.eg.db v3.20.0, JASPAR database, ChIP-Atlas database, freebayes v1.3.9, samtools/htslib v1.19, bcftools v1.14, VariantAnnotation v1.52.0, biomaRt v2.61.1, tidyverse suite v2.0.0, R v4.3, Python 3.10.7

For manuscripts utilizing custom algorithms or software that are central to the research but not yet described in published literature, software must be made available to editors and reviewers. We strongly encourage code deposition in a community repository (e.g. GitHub). See the Nature Portfolio guidelines for submitting code & software for further information.

Data

Policy information about availability of data

All manuscripts must include a data availability statement. This statement should provide the following information, where applicable:

- Accession codes, unique identifiers, or web links for publicly available datasets
- A description of any restrictions on data availability
- For clinical datasets or third party data, please ensure that the statement adheres to our policy

The mouse RRM-KO, lacZ, and rat data generated in this study have ben deposited in the European Nucleotide Archive (ENA) at EMBL-EBI under accession code PRJEB89457 (https://www.ebi.ac.uk/ena/browser/view/PRJEB89457). In addition we analyzed the following publicly available datasets: DCM and Control samples available in European Genome-Phenome Archive und accession code EGAD00001003390 (https://ega-archive.org/datasets/EGAD00001003390) and EGAD00001003391 (https://ega-archive.org/datasets/EGAD00001003391), HCM and Control samples available on the NIH Gene Expression Omnibus (GEO) under the accession number GSE249925 (https://www.ncbi.nlm.nih.gov/geo/query/acc.cgi?acc=GSE249925), and the mouse developmental data deposited in the GEO repository under the accession number GEO: GSE213233 (https://www.ncbi.nlm.nih.gov/geo/query/acc.cgi?acc=GSE213233). Source Data are provided with this paper.

Research involving human participants, their data, or biological material

Policy information about studies with human participants or human data. See also policy information about sex, gender (identity/presentation), and sexual orientation and race, ethnicity and racism.

Reporting on sex and gender	We refer to sex as biological sex and do not refer to gender.
Reporting on race, ethnicity, or other socially relevant groupings	N/A
Population characteristics	N/A
Recruitment	N/A
Ethics oversight	N/A

Note that full information on the approval of the study protocol must also be provided in the manuscript.

Field-specific reporting

Please select the one below that is the best fit for your research. If you are not sure, read the appropriate sections before making your selection.

☒ Life sciences ☐ Behavioural & social sciences ☐ Ecological, evolutionary & environmental sciences

For a reference copy of the document with all sections, see nature.com/documents/nr-reporting-summary-flat.pdf

Life sciences study design

All studies must disclose on these points even when the disclosure is negative.

Sample size	For animal experiments, we use G*power for power calculation with submitted with the animal protocol application. For work on tissue samples, we routinely use at least 3 animals per group.
Data exclusions	Outliers were identified using the interquartile range (IQR) method, defining values as outliers if they fell below the first quartile minus 1.5 times the IQR or above the third quartile plus 1.5 times the IQR.
Replication	For animal and cell culture experiments, we use at least three biological replicates. Replicates were successful.
Randomization	We randomized animals to experimental groups where applicable.
Blinding	We blinded investigators during acquisition and analysis for echocardiography and catheter measurements.

Reporting for specific materials, systems and methods

We require information from authors about some types of materials, experimental systems and methods used in many studies. Here, indicate whether each material, system or method listed is relevant to your study. If you are not sure if a list item applies to your research, read the appropriate section before selecting a response.

Materials & experimental systems

n/a	Involved in the study	
☐	☒	Antibodies
☐	☒	Eukaryotic cell lines
☒	☐	Palaeontology and archaeology
☐	☒	Animals and other organisms
☐	☒	Clinical data
☒	☐	Dual use research of concern
☒	☐	Plants

Methods

n/a	Involved in the study	
☒	☐	ChIP-seq
☒	☐	Flow cytometry
☒	☐	MRI-based neuroimaging

Antibodies

Antibodies used	All antibodies are listed in supplementary table 2 and 3
Validation	Antibodies are routinely validated if they do not produce expected results on positive controls in Western and/or IF.

Eukaryotic cell lines

Policy information about cell lines and Sex and Gender in Research

Cell line source(s)	HEK and HEK EBMA from
Authentication	N/A
Mycoplasma contamination	routinely tested, negative
Commonly misidentified lines (See ICLAC register)	N/A

Animals and other research organisms

Policy information about studies involving animals; ARRIVE guidelines recommended for reporting animal research, and Sex and Gender in Research

Laboratory animals	animals were backcrossed on a 129/S6 and only male mice were used at about 100 days of age. Adult rat BN and SHR strains.
Wild animals	N/A
Reporting on sex	N/A
Field-collected samples	N/A
Ethics oversight	All experiments involving animals were performed according to institutional guidelines and had been approved by the local authorities (LAGeSo Berlin).

Note that full information on the approval of the study protocol must also be provided in the manuscript.

Clinical data

Policy information about clinical studies

All manuscripts should comply with the ICMJE guidelines for publication of clinical research and a completed CONSORT checklist must be included with all submissions.

Clinical trial registration	N/A
Study protocol	N/A
Data collection	N/A
Outcomes	N/A

Plants

Seed stocks	N/A
Novel plant genotypes	N/A
Authentication	N/A
